# Management of ocular involvement in the acute phase of Stevens-Johnson syndrome and toxic epidermal necrolysis: french national audit of practices, literature review, and consensus agreement

**DOI:** 10.1186/s13023-020-01538-x

**Published:** 2020-09-22

**Authors:** D. Thorel, S. Ingen-Housz-Oro, G. Royer, A. Delcampe, N. Bellon, C. Bodemer, A. Welfringer-Morin, D. Bremond-Gignac, M. P. Robert, M. Tauber, F. Malecaze, O. Dereure, V. Daien, A. Colin, C. Bernier, C. Couret, B. Vabres, F. Tetart, B. Milpied, T. Cornut, B. Ben Said, C. Burillon, N. Cordel, L. Beral, N. de Prost, P. Wolkenstein, M. Muraine, J. Gueudry

**Affiliations:** 1 Département d’Ophtalmologie, CHU Charles Nicolle, F-76000 Rouen, France; 2 Centre de référence des dermatoses bulleuses toxiques et toxidermies graves TOXIBUL, Créteil, France; 3 Département de Dermatologie, AP-HP, Hôpital Henri Mondor, 51 avenue du maréchal de Lattre de Tassigny, 94000 Créteil, France; 4 EA7379 EpidermE, Créteil, France; 5 Département d’Ophtalmologie, AP-HP, Hôpital Henri Mondor, Créteil, France; 6 Département de Dermatologie, AP-HP, Hôpital Necker, Paris, France; 7 Département d’Ophtalmologie, AP-HP, Hôpital Necker, Paris, France; 8 Département de Dermatologie, CHU Toulouse, Toulouse, France; 9 Département d’Ophtalmologie, CHU Toulouse, Toulouse, France; 10 Département de Dermatologie, Université de Montpellier et INSERM U1058 Pathogenèse et contrôle des infections chroniques, CHU Montpellier, Montpellier, France; 11 Département d’Ophtalmologie, CHU Montpellier, Montpellier, France; 12 Département de Dermatologie, CHU Nantes, Nantes, France; 13 Département d’Ophtalmologie, CHU Nantes, Nantes, France; 14 Département de Dermatologie, CHU Charles Nicolle, F-76000 Rouen, France; 15 Département de Dermatologie, CHU Saint-André, Bordeaux, France; 16 Département d’Ophtalmologie, CHU Pellegrin, Bordeaux, France; 17 Département de Dermatologie, Hôpital Edouard Herriot, Lyon, France; 18 Département d’Ophtalmologie, Hôpital Edouard Herriot, Lyon, France; 19 Unité de Dermatologie et d’Immunologie clinique, CHU Guadeloupe, Pointe-à-Pitre, Guadeloupe France; 20 Département d’Ophtalmologie, CHU Guadeloupe, Pointe-à-Pitre, Guadeloupe France; 21 Réanimation médicale, AP-HP, Hôpital Henri Mondor, Créteil, France

**Keywords:** Stevens-Johnson syndrome, Lyell syndrome, Toxic epidermal necrolysis, Management, Ocular involvement, Treatment, Drug reaction, Eye

## Abstract

Stevens-Johnson Syndrome (SJS) and toxic epidermal necrolysis (TEN) can lead to severe ophthalmologic sequelae. The main risk factor is the severity of the initial ocular involvement. There are no recommendations for ocular management during acute phase.

We conducted a national audit of current practice in the 11 sites of the French reference center for toxic bullous dermatoses and a review of the literature to establish therapeutic consensus guidelines. We sent a questionnaire on ocular management practices in SJS/ TEN during acute phase to ophthalmologists and dermatologists. The survey focused on ophthalmologist opinion, pseudomembrane removal, topical ocular treatment (i.e. corticosteroids, antibiotics, antiseptics, artificial tear eye drops, vitamin A ointment application), amniotic membrane transplantation, symblepharon ring use, and systemic corticosteroid therapy for ophthalmologic indication. Nine of 11 centers responded. All requested prompt ophthalmologist consultation. The majority performed pseudomembrane removal, used artificial tears, and vitamin A ointment (8/9, 90%). Combined antibiotic-corticosteroid or corticosteroid eye drops were used in 6 centers (67%), antibiotics alone and antiseptics in 3 centers (33%). Symblepharon ring was used in 5 centers (55%) if necessary. Amniotic membrane transplantation was never performed systematically and only according to the clinical course. Systemic corticosteroid therapy was occasionally used (3/9, 33%) and discussed on a case-by-case basis.

The literature about ocular management practice in SJS/ TEN during acute phase is relatively poor. The role of specific treatments such as local or systemic corticosteroid therapy is not consensual. The use of preservatives, often present in eye drops and deleterious to the ocular surface, is to be restricted. Early amniotic membrane transplantation seems to be promising.

## Background

Stevens-Johnson syndrome (SJS) and toxic epidermal necrolysis (TEN, or Lyell syndrome) are serious and rare diseases, most often drug-induced, and their incidence has recently been re-estimated at 6 cases/million/year in France [1]. SJS and TEN are characterized by widespread necrosis of the epidermis and the mucous membrane, sometimes complicated during the acute phase by multi-organ failure that can be fatal. They differ according to the extent of skin detachment they cause, SJS < 10%, TEN or Lyell syndrome ≥30%, and SJS/TEN overlap 10–29% [2]. In the acute phase, patients often have widespread, variable-degree mucosal involvement, particularly in the eyes (80%) [3, 4]. Ocular involvement is classified, according to Power, into three categories [3]. The mild degree consists in mild lid edema, and/or conjunctival injection, and/or chemosis only. The moderate degree consists in pseudomembranous conjunctivitis, and/or keratitis healing with medical treatment, and/or corneal ulceration, and/or corneal infiltrates. The severe degree consists in the formation of symblepharon, persistent non-healing keratitis despite medical treatment, and/or decrease in visual acuity, and/or conjunctival fornix foreshortening. Another classification has been proposed by Sotozono et al. [5]. The overall mortality of the acute phase is around 15% [6]. Most survivors have more or less severe sequelae, usually cutaneous, ocular, and psychological, with long-term impact on the quality of life [7]. Twenty to 79% of patients present with potentially vision-threatening chronic ocular manifestations [8]. The main risk factors of severe long-term ocular sequelae are the severity of the initial ocular lesions and the overall severity of the acute phase [9, 10]. Moreover, skin phototypes V and VI have recently been reported as an additional risk factor for more severe, long-term ocular complications [11].

Owing to the lack of effective immunomodulatory treatment that can reduce mortality and muco-cutaneous scarring, the main treatment in the acute phase is symptomatic or supportive care [1]. So far, there has been no standardized protocol and no consensus in the literature.

We conducted a national audit of ophthalmologic management practices during the acute phase of SJS/TEN in the 11 sites of the French Reference Center for Toxic Bullous Dermatoses, and compared our results with those of the literature. Thanks to this audit, we were able to establish suggestions for standardized collection of clinical data and management of ocular involvement during the acute phase.

## Methods

First, we audited the practices used in the 11 dermatology departments of the Reference Center for Toxic Bullous Dermatoses in France. In an e-mail sent to each referent dermatologist, we enclosed an 11-item questionnaire detailing the practices used to provide ophthalmologic care during the acute phase of SJS/TEN. Dermatologists had to answer the questionnaire in collaboration with the referent ophthalmologist.

The second step was to perform a literature review. We searched PubMed for all articles published between 1994 and 2019, which analyzed ocular management during the acute phase of SJS/TEN. Articles focused on management of late ocular sequelae or the impact of systemic treatments on other non-ocular parameters were excluded. The bibliographic search was guided by three themes: type of local drug administration (i.e. topical corticosteroid, artificial tears, vitamin A ointment, and topical antibiotics), type of systemic treatment (i.e. systemic steroid therapy, intravenous immunoglobulin (IV-Ig), and cyclosporine), and type of adjuvant treatment and/or ophthalmologic surgery such as amniotic membrane transplantation (AMT).

## Results

### Audit of practices

Nine dermatology departments (80%) replied to the questionnaire (Table 1). All nine highlighted the necessity of performing rapid ophthalmologic evaluation and continuous follow-up. Two centers (22%) had a well-established ophthalmology-dermatology protocol. Sterile preservative-free saline ocular irrigation was performed in both centers. In the first center, vitamin A ointment was applied abundantly every 2 to 4 h. In the second center, antiseptic eye drops (picloxydine 0.05% eye drops) and preservative-free artificial tears were instilled every 2 h. Mechanical removal of pseudomembranes was performed in both centers if necessary. Both centers focused on regular clinical follow-up with the ophthalmologist team. Pseudomembrane removal, and application of artificial tears and/or vitamin A ointment are routine practice in most centers (8/9, 90%). Combined antibio-corticosteroid (dexamethasone/tobramycin, dexamethasone/neomycin) eye drops were administered in six centers (67%). Antiseptic eyedrops (picloxydine eye drops) or antibiotic eye drops (ciprofloxacin or rifamycin eye drops) were used less commonly (3/9, 33%). Symblepharon rings were used in five centers (55%), but never as a first option. Systemic corticosteroid therapy was used on a case-by-case basis (3/9, 33%). No other systemic treatment with ocular indication was used. In all centers, AMT was performed in operating room on a case-by-case basis.

**Table 1:** Audit of current ocular management during acute phase of SJS/Lyell in the 11 sites of the French reference center for toxic bullous dermatoses

	9 answers, n/9 (%)
**Standardised ophthalmologic protocols**	2 (22)
**Pseudomembrane removal**	8 (90)
**Application of eye drops:**	
Corticosteroid/combined antibio-corticosteroid	6 (67)
Antiseptic/antibiotic alone	3 (33)
Artificial tears	8 (90)
**Vitamin A ointment**	8 (90)
**Systemic corticosteroid therapy**	3 (33)
**Symblepharon rings**	5 (55)
**Amniotic membrane transplantation on a case-by-case basis**	9 (100)

### Literature review

We selected 13 articles as shown in Table 2. Most studies were retrospective and conducted on relatively small samples.

- Topical ocular treatment

**Table 2:** Literature review focus on ocular management during acute phase of SJS/Lyell

Treatments	Author/article [Ref]	Year/country	Study methodology	Number patients (n)	Conclusion
**Topical treatment**					
Topical corticosteroid therapy	Sotozono et al. [5]	2009/ Japan	Retrospective controlled	94	Improvement of visual prognosis
Topical antibiotics	Yip et al. [12]	2007/ Singapore	Retrospective	117	Increased risk of ocular complications
Antibiotics/corticosteroids/antiseptics	Gueudry et al. [9]	2009/ France	Retrospective	159	No impact on ocular complications
**Systemic treatment**					
Systemic corticosteroid therapy	Power et al. [3]	1995/USA	Retrospective controlled	366	At 3 months, no significant difference in ocular involvement
IV-Ig	Yip et al. [13]	2005/Singapore	Retrospective controlled	10	No significant difference in ocular complications between patients treated with 2-day IV-Ig (2 g/kg)
IV-Ig	Kim et al. [14]	2013/Korea	Retrospective comparative multicentric	51	An early high-dose IV-Ig or systemic steroid could improve VA on the long term
Systemic and topical corticosteroid therapy	Araki et al. [15]	2009/Japan	Observational prospective	5	No late ocular complications in patients treated with corticosteroid pulse (500 mg - 1 g for 3 days) + topical corticosteroid
Systemic corticosteroid/ IV-Ig/ combined corticosteroid IV-Ig therapy/ supportive care only (combined antibio-corticosteroid eye drops, artificial tears)	Kim et al. [16]	2015/Korea	Retrospective multicentric comparative	43	No significant difference between groups of patients treated with IV-Ig or systemic steroid or supportive care
**Adjuvant treatment**					
AMT	Sharma et al. [17]	2016/India	Randomised controlled trial	50	Improvement of tear film break up time, visual acuity, Schirmer’s test, and reduction of conjunctival inflammation at 6 months
AMT/Self-retained AMT	Gregory [18]	2011/USA	Observational prospective non-controlled	10	Decreased palpebral inflammation and symblepharon formation, lower incidence of late ocular complications at 6 months in patients treated with AMT or self-retained AMT
AMT/Self-retained AMT	Shanbhag et al. [19]	2019/USA	Controlled retrospective observational	48	Reduced ocular complications and improved final VA in patients with early AMT or self-retained AMT
AMT	Gregory [20]	2016/USA	Observational prospective non-controlled	79	Improvement of VA and decreased dry eye symptoms or scarring sequelae
AMT	Shammas et al. [21]	2010/USA	Observational retrospective	6	Acute phase AMT combined with topical corticosteroids resulted in better VA and ocular surface preservation

*AMT* amniotic membrane transplantation; *IV-Ig* intravenous immunoglobulins; *VA* visual acuity

Three articles were selected on the use of topical eye drops during the acute phase of SJS/TEN. Sotozono et al showed improvement in visual acuity upon using topical corticosteroid in the first week after disease onset [5]. Yip et al. demonstrated a significantly increased risk of ocular complications in patients treated during the acute phase with topical antibiotics, such as chloramphenicol or tetracycline, alone or combined, or with eye drops containing preservatives, such as thiomersal and phenylmercuric nitrate [12]. Gueudry et al. studied the impact of topical therapies during the acute phase, such as artificial tears, antiseptic eye drops, antibiotic eye drops, or combined antibio-corticosteroid eye drops on the prevention of late ocular complications. No significant difference was found. The presence of preservatives such as benzalkonium chloride in these eye drops was not associated with higher risk of ocular complications [9].

- Systemic treatment

Five articles were selected in this category. Power et al. found that systemic steroid therapy (prednisolone, mean dose 54 mg/day) used during the acute phase did not significantly change the outcome in terms of ocular involvement at 3 months of treatment [3]. All patients had concomitant topical treatment (antibiotics, corticosteroids, artificial tears). A recent study compared systemic corticosteroid therapy, IV-Ig therapy, combined corticosteroid IV-Ig therapy, and topical treatments only (combined antibio-corticosteroid eye drops and artificial tears). No significant difference in final visual acuity or ocular complication score was detected [16]. Yip et al. retrospectively studied a small group of patients treated with IV-Ig (2 g/kg) and compared them with a historical cohort of IV-Ig non-treated patients and found no significant difference in ocular complications [13]. Conversely, Kim et al. showed that early high dose IV-Ig (2.7 g/kg) or high dose systemic corticosteroids (mean 5.3 mg/kg/day) could improve visual acuity in the long term [14, 16]. Araki et al. conducted a prospective study to evaluate the impact of using systemic intravenous corticosteroid therapy (500 mg to 1 g/day for 3 days) + topical corticosteroid (5 times a day). At inclusion, half of the patients had severe ocular involvement with corneal ulceration. After 1 year, they had no ocular sequelae and their visual acuity was 20/20 (5 patients) [15].

- Adjuvant ophthalmologic treatment

Five articles on AMT and other specific ophthalmologic procedures used during the acute phase of SJS/TEN were selected. A single randomized controlled trial conducted by Sharma et al. showed improvement in tear film break-up time, visual acuity, Schirmer’s test and also a reduction in conjunctival inflammation after 6 months of AMT. The control group received standard topical treatment (i.e. chloramphenicol 0.5% and polymyxin B sulphate eye drops, corticosteroid eye drops, artificial tears, and surgical debridement). AMT was performed at bedside followed by the insertion of a symblepharon ring [17]. Other studies on AMT had no control group. Gregory reported a sample of 10 patients treated with AMT within 10 days from disease onset. Three patients were treated with a self-retained AMT (Prokera®, Bio-Tissue, Inc), consisting in a sutureless insertion. At 6 months, patients had reduced palpebral inflammation, reduced symblepharon size, and lower incidence of late ocular complications [18]. More recently, Shanbhag et al. demonstrated an improvement in final visual acuity and a reduction in ocular complications in patients treated with early AMT or the self-retained AMT compared to those treated medically alone (antibiotic eye drops, corticosteroid eye drops, etc.) [19]. Gregory et al. evaluated in their observational study the usefulness of AMT within the first 10 days of hospitalization for very severe cases [20]. AMT was repeated every 10–14 days, as long as there was ocular inflammation, and resulted in improvement of visual acuity and ocular surface preservation. AMT combined with topical corticosteroids could be useful, as reported by Shammas et al. [21].

Surgical debridement using cotton-tip applicator is poorly analyzed, hence the absence of evaluation in the literature. We found no studies on the use of symblepharon ring alone.

## Conclusions

Our national audit of medical practices in France reveals that ophthalmologic management during the acute phase of SJS/TEN relies mainly on early evaluation by an ophthalmologist, surgical debridement of pseudomembranes, and application of artificial tears and/or vitamin A eye ointment. A relative trend was observed for a consensus agreement in clinical practices despite the poor evidence in the literature.

**Fig. 1:**
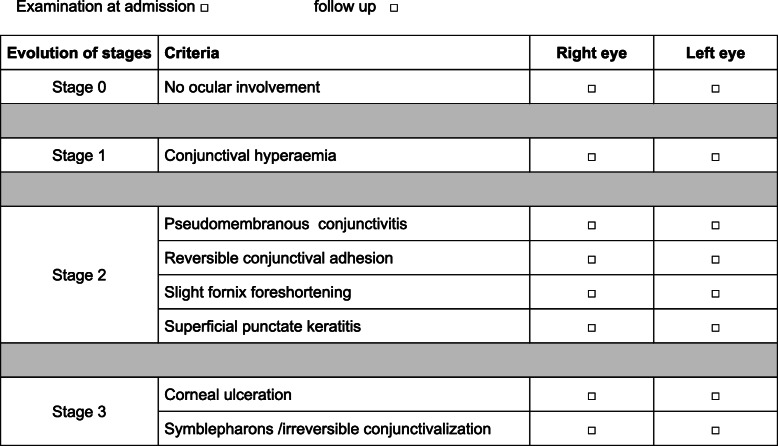
Diagnostic evaluation of acute-phase ocular involvement

This audit has provided us with a base to propose a consensual diagnostic evaluation during acute phase consisting in three stages of severity (Fig. 1). This evaluation is based on the classification of Power [3], in which persistent corneal ulceration is an important indicator of severity, and on that of Sotozono et al. [5]. For long-term hospitalization or mild cases, we added other criteria such as formation of symblepharons, which are more visible towards the end of the acute phase, and superficial punctate keratitis, which can be seen by slit-lamp examination after fluorescein staining.

**Fig. 2:**
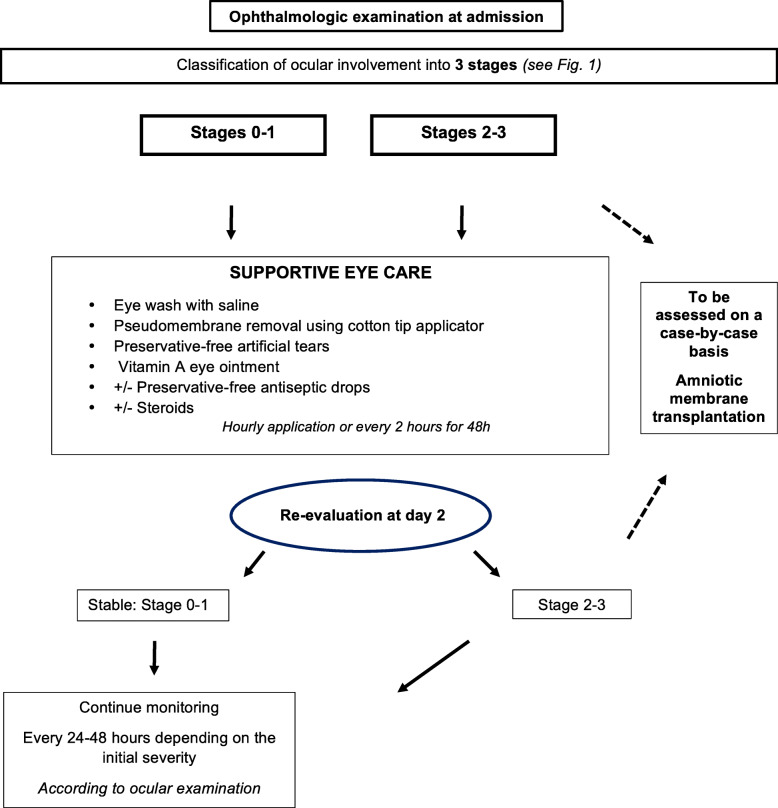
Acute-phase ocular management proposition in patients with Stevens-Johnson syndrome and toxic epidermal necrolysis

Considering our audit results and literature review, we propose therapeutic recommendations for ocular management in the acute phase of SJS/TEN that should be adjusted to each patient (Fig. 2). The use of artificial tears with high wetting power and no preservatives and vitamin A eye ointment seems to be strongly recommended. Lubricating the eye is vital to maintain and improve tear film quality [22]. Nonetheless, the interest of using artificial tears in the acute phase has not been supported by solid evidence. Applying antibiotic eye drops to prevent infections on altered ocular surface may lead to side-effects, which could be explained by two hypotheses: direct toxicity of preservatives on ocular surface or hypersensitivity to antibiotic eye drops [12]. As a consequence, we advise using preservative-free artificial tears or vitamin A eye ointment to provide optimum protection of ocular surface. We do not support the use of prophylactic antibiotic eye drops, even though it is widely practised. There is no evidence in the literature in favor of using systemic corticosteroids for the prevention of ocular complications. Topical corticosteroid therapy might provide such prevention, even though its use remains controversial [23].

AMT could represent the most promising procedure and should be considered to treat severe cases to protect the ocular surface. In the literature, a case-report suggested that AMT helps the corneal epithelium to heal rapidly and reduces ocular surface inflammation, when the procedure is performed in the first 2 weeks of disease onset [24]. However, AMT, during the acute phase, in patients with extensive skin detachment, sometimes in intensive care unit with mechanical ventilation, is hindered by some medical and practical issues. Self-retained AMT, relies on a recent device, not commercially available in France, composed of an amniotic membrane patch preloaded on symblepharon ring, and which can be easily inserted at bedside and with no surgical suturing. This biological patch covers the ocular surface and prevents it from coming into contact with the eyelids, and thus reduces adhesions. Similarly, there are no studies on acute phase surgical debridement or its usefulness in preventing late ocular complications, however, largely carried out.

In France, ocular management during the acute phase of SJS/TEN mainly relies on pseudomembrane removal, application of preservative-free artificial tears with high wetting power and vitamin A eye ointment after rapid ophthalmologist consultation. The main objective is to preserve the ocular surface. AMT is performed under certain conditions and according to clinical evolution. There is a relatively high level of evidence towards the effectiveness of AMT in the literature. The use of eye drops containing preservatives should be spared. Given that ocular sequelae are the main long-term complications of SJS/TEN, studies are warranted in the future to explicitly define the optimum ocular management during acute phase.

## Data Availability

The data are available on request to the corresponding author.

## Abbreviations

SJS: Stevens-Johnson Syndrome
TEN: Toxic Epidermal Necrolysis
IV-Ig: Intravenous immunoglobulin
AMT: Amniotic Membrane Transplantation
